# Ubiquinol reduces gamma glutamyltransferase as a marker of oxidative stress in humans

**DOI:** 10.1186/1756-0500-7-427

**Published:** 2014-07-04

**Authors:** Simone Onur, Petra Niklowitz, Gunnar Jacobs, Ute Nöthlings, Wolfgang Lieb, Thomas Menke, Frank Döring

**Affiliations:** 1 Institute of Human Nutrition and Food Science, Division of Molecular Prevention, Christian Albrechts University Kiel, Heinrich-Hecht-Platz 10, 24118 Kiel, Germany; 2 Children’s Hospital of Datteln, University of Witten/Herdecke, Dr.-Friedrich-Steiner Str. 5, 45711 Datteln, Germany; 3 Institute of Epidemiology and Biobank Popgen, Christian Albrechts University Kiel, Campus University Hospital Schleswig-Holstein, Niemannsweg 11, Haus 1, 24105 Kiel, Germany; 4 Institute of Nutrition and Food Science, Rheinische Friedrich Wilhelms University Bonn, Meckenheimer Allee 168, 53115 Bonn, Germany

**Keywords:** Coenzyme Q_10_, Ubiquinol, Gene expression, Supplementation study, Liver enzymes, Oxidative stress, Antioxidants

## Abstract

**Background:**

The reduced form of Coenzyme Q_10_ (CoQ_10_), ubiquinol (Q_10_H_2_), serves as a potent antioxidant in mitochondria and lipid membranes. There is evidence that Q_10_H_2_ protects against oxidative events in lipids, proteins and DNA. Serum gamma-glutamyltransferase (GGT) activity is associated with cardiovascular diseases. In a physiological range, activity of GGT is a potential early and sensitive marker of inflammation and oxidative stress.

In this study, we first examined the relationship between CoQ_10_ status and serum GGT activity in 416 healthy participants between 19 and 62 years of age in a cross-sectional study (cohort I). In the second step, 53 healthy males (21–48 years of age; cohort II) underwent a 14-day Q_10_H_2_ supplementation (150 mg/d) to evaluate the effect of Q_10_H_2_ supplementation on serum GGT activity and *GGT1* gene expression.

**Findings:**

There was a strong positive association between CoQ_10_ status and serum GGT activity in cohort I. However, a gender-specific examination revealed differences between male and female volunteers regarding the association between CoQ_10_ status and serum GGT activity. Q_10_H_2_ supplementation (cohort II) caused a significant decrease in serum GGT activity from T_0_ to T_14_ (p < 0.001). *GGT1* mRNA levels declined 1.49-fold after Q_10_H_2_ supplementation. Of note, other liver enzymes (i.e., aspartate aminotransferase, AST) were not affected by Q_10_H_2_ supplementation.

**Conclusions:**

CoQ_10_ level is positively associated with serum GGT activity. Supplementation with Q_10_H_2_ reduces serum GGT activity. This effect might be caused by gene expression. Overall, we provide preliminary evidence that higher Q_10_H_2_ levels improve oxidative stress via reduction of serum GGT activity in humans.

**Trial registration:**

Current Controlled Trials ISRCTN26780329.

## Background

Coenzyme Q_10_ (CoQ_10_) is a redox molecule that is present in the membranes of almost all human tissues [1]. CoQ_10_ is a lipophilic molecule that is synthesized within the mitochondrial inner membrane and is essential for the respiratory transport chain. As an antioxidant in cell membranes, CoQ_10_ is important for the maintenance of the cellular redox homeostasis [2]. Furthermore, CoQ_10_ is necessary for pyrimidine biosynthesis while also being a cofactor for uncoupling proteins [3]. It has also been identified as a modulator of gene expression [4-6], inflammatory processes [7-9] and apoptosis [10,11]. The reduced form of CoQ_10_, ubiquinol (Q_10_H_2_), serves as a potent antioxidant in mitochondria and lipid membranes as well as a regenerator of other lipid soluble antioxidants [12]. In recent years, there has been growing evidence of the protective role of Q_10_H_2_ in oxidative events in lipids, proteins and DNA, a concept known as oxidative stress [13,14].

The liver enzyme gamma glutamyltransferase (GGT) is located at the outer surface of the plasma membrane where it facilitates the synthesis of the antioxidant glutathione (GSH) [15]. GGT initiates the degradation of extracellular GSH by hydrolyzing the γ-glutamyl-cysteine bond in GSH [16]. The regulation of GGT is complex. Human GGT is encoded by a multi-gene family consisting of at least seven genes. One of these genes, *GGT1*, encodes for the active enzyme present in human tissues [17]. Serum GGT has long been regarded as a marker for excessive alcohol consumption or liver disturbances in clinical practice [18]. However, it has recently been shown that serum GGT is also a marker for the development of cardiovascular disease, hypertension, stroke and type 2 diabetes mellitus and their complications independent from alcohol consumption [19-22]. Further studies provide evidence that serum GGT activity is a potential early and sensitive marker of inflammation and oxidative stress [23-25]. Of note, this effect was only found in the physiological range of GGT activity.

It is known that oxidative stress can be attenuated by Q_10_H_2_[13,14]. Because serum GGT is a putative marker of oxidative stress, we examined (i) the relationship between CoQ_10_ status and serum GGT activity in a sample of 416 healthy volunteers (cohort I) and (ii) the effect of Q_10_H_2_ supplementation on serum GGT activity and *GGT1* gene expression in an intervention cohort of 53 healthy male volunteers (cohort II).

## Methods

### Study populations

#### Cohort I – cross-sectional study

The study sample was part of the PopGen control cohort. The original study design is described elsewhere [26]. The study population consisted of 416 healthy blood donors between 19 and 62 years old with a mean body mass index (BMI) of 26.1 kg/m^2^ (standard deviation, 4.7 kg/m^2^). A total of 53% were males. All study participants were phenotyped with respect to anthropometric, cardiovascular and metabolic traits in a standardized fashion (details below). Blood samples were taken after an overnight fast and immediately centrifuged. Serum samples were stored at -80°C until CoQ_10_ was analyzed. The participants had no history of gastrointestinal, hepatic, cardiovascular or renal diseases; maintained usual nutrition habits; and were non-smokers or occasional smokers. The study was approved by the ethics committee of the Medical Faculty of Kiel University and was consistent with the Helsinki Declaration. All volunteers gave written informed consent. Recruitment of study probands as well as data and biosample collection were performed using the PopGen biobank, Kiel, Germany.

#### Cohort II – supplementation study

The sample characteristics and study design have been described previously [27]. Briefly: 53 healthy male volunteers between 21 and 48 years of age received 150 mg of the reduced form of CoQ_10_ (Q_10_H_2_, ubiquinol, KANEKA Corporation, Japan) daily in the form of three capsules à 50 mg Q_10_H_2_ taken with each main meal (breakfast, lunch and dinner) for 14 days. Fasting blood samples were taken before (T_0_) and after (T_14_) supplementation. The participants had an average BMI of 24.1 ± 2.5 kg/m^2^; had no history of gastrointestinal, hepatic, cardiovascular or renal diseases; maintained usual nutrition habits; and were non-smokers or occasional smokers. The study was approved by the ethics committee of the Medical Faculty of Kiel University, Germany, and was consistent with the Helsinki Declaration. All volunteers gave written informed consent.

### CoQ_10_ analysis

CoQ_10_ analysis was based on the method of high-pressure liquid chromatography (HPLC) with electrochemical detection and internal standardization using ubihydroquinone-9 and ubiquinone-9 as standards, as described elsewhere [28]. 100 μL plasma aliquots were used in both cohorts to perform robust HPLC analysis with repeats, when indicated.

### Anthropometric measurements

Body weight was measured in underwear on a manual scale to the nearest 100 g (Seca, Hamburg, Germany). Height was measured without shoes on a stadiometer (Seca, Hamburg, Germany) to the nearest 0.5 cm.

### Clinical parameters, metabolic parameters and gene expression

Blood pressure measurements were obtained while the subject was in a seated position, using a standard manual sphygmomanometer. A fasting venous blood sample was obtained from all study participants and analyzed following standard procedures. Briefly, blood glucose was analyzed using a hexokinase method (Gluco-quant, Roche Diagnostics, Mannheim, Germany). Cholesterol and triacylglycerol concentrations were measured enzymatically by hydrolyzing cholesterol ester and triacylglycerol to cholesterol and glycerol, respectively. HDL cholesterol (HDL-C) was measured in the supernatant after precipitation of lipoproteins (kits and standards by Konelab Corporation, Espoo, Finland). The liver enzymes gamma glutamyltransferase (GGT) and aspartate aminotransferase (AST) were analyzed according to the recommendations of the International Federation of Clinical Chemistry (IFCC) from 1983 (confirmed and extended in 2002), including optimization of substrate concentrations and employment of NaOH, glycylglycine buffer and sample start. Plasma C-reactive protein (CRP) concentrations were measured using a latex enhanced nephelometric assay run on a BN II hematology analyzer (Dade Behring, Marburg, Germany). Microarray experiments using the Affymetrix human genome U133 plus 2.0 GeneChip® were performed as previously described [5] with RNA samples from CD14-positive monocytes obtained from three volunteers before (T_0_) and after (T_14_) supplementation with ubiquinol.

### Statistics

Statistical analyses were performed with SPSS (Statistical Package for the Social Sciences) 20.0 software (SPSS GmbH Software, München, Germany). Before statistical analysis, normal distribution of the parameters was tested. Gender-related differences in anthropometric and metabolic parameters and differences between subjects with normal and elevated GGT activity were analyzed using a nonparametric Mann–Whitney *U*-test. Differences from supplementation regarding two time-points (T_0_ vs. T_14_) were tested using a paired Student’s *t*-test. The level of statistical significance was set at p < 0.05.

## Findings

### Study population – cohort I

**Figure 1:**
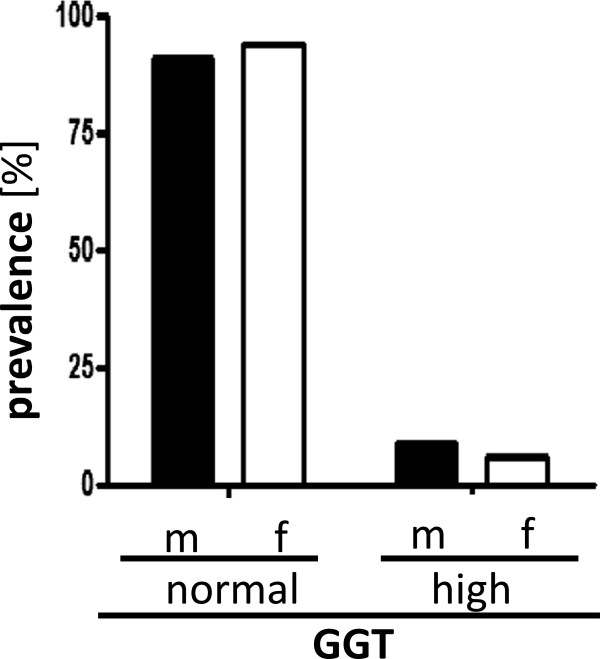
Prevalence of elevated gamma glutamyltransferase (GGT) activity levels (≥50/40 U/l m/f) in males (m; n = 220) and females (f; n = 196).

We examined 416 healthy volunteers in a cross-sectional study. As shown in Figure 1, more than 90% of the study population had normal activity of serum GGT (<50/40 U/l male/female), whereas 9.1% (n = 21) of male and 6.1% (n = 12) of female volunteers had increased serum GGT activity. Because serum GGT activity is postulated to be a marker of oxidative stress in its normal range, study subjects with elevated serum activity of GGT were excluded.

The characteristics of cohort I are depicted in Table 1. Study subjects were healthy but slightly overweight (mean BMI 26.2 ± 4.7 kg/m^2^) with a mean age of 40.0 years. On average, values of CoQ_10_ status and pro-inflammatory CRP were in a normal range. Components of metabolic syndrome were below the thresholds defined by the Adult Treatment Panel III (ATPIII). Gender-stratified analyses revealed significant differences between males and females. Compared to women, men had higher BMI, total CoQ_10_, ubiquinol, GGT activity, blood pressure, triglycerides, total and LDL-cholesterol and glucose, whereas women had higher CRP- and HDL-cholesterol levels than men. Additionally, female volunteers tended to have a higher CoQ_10_-redox state (% oxidized CoQ_10_ in total CoQ_10_; p = 0.054) compared to males.

**Table 1:** Basic characteristics of cohort I (subsample of the popgen control cohort)

	**All (n = 383)**	**Males (n = 199)**	**Females (n = 184)**
**Age [years]**	40.0 ± 10.9	40.5 ± 10.5	38.7 ± 11.2
**Body mass index [kg/m**^**2**^**]**	26.2 ± 4.7	26.2 ± 3.9	25.7 ± 5.2**
**CoQ**_**10 **_**[μmol/l]**	0.94 ± 0.34	0.96 ± 0.36	0.87 ± 0.29*
**Ubiquinol [μmol/l]**	0.12 ± 0.05	0.12 ± 0.04	0.11 ± 0.04
**Ubiquinone [μmol/l]**	0.82 ± 0.30	0.84 ± 0.33	0.76 ± 0.25*
**CoQ**_**10 **_**redox state [%]**	12.5 ± 2.6	12.2 ± 2.5	12.7 ± 2.7
**Gamma GT [U/l]**	18.8 ± 8.4	22.5 ± 8.3	14.7 ± 6.3***
**C-reactive protein[mg/l]**	2.24 ± 3.36	1.58 ± 1.92	2.47 ± 3.00***
**Systolic BP [mm Hg]**	130 ± 16	134 ± 15	125 ± 14***
**Diastolic BP [mm Hg]**	78 ± 9	80 ± 9	75 ± 8***
**Triglycerides [mg/dl]**	121.9 ± 73.3	132.1 ± 76.1	100.5 ± 50.3***
**Cholesterol [mg/dl]**	193.3 ± 35.7	194.3 ± 39.8	190.3 ± 28.8
**LDL-cholesterol [mg/dl]**	116.6 ± 32.5	122.6 ± 34.7	108.0 ± 26.1***
**HDL-cholesterol [mg/dl]**	62.1 ± 16.7	54.8 ± 13.4	71.1 ± 16.1***
**Glucose [mg/dl]**	92.5 ± 10.7	94.1 ± 11.3	90.1 ± 9.7***

CoQ_10_ = Coenzyme Q_10_, Gamma GT = gamma glutamyltransferase; BP = blood pressure; LDL = low density lipoprotein, HDL = high density lipoprotein.

Data are presented as mean ± SD; *p < 0.05; **p < 0.01; ***p < 0.001 significant differences between sexes, Mann–Whitney U-test.

### CoQ_10_ is related to serum GGT activity by gender

**Figure 2:**
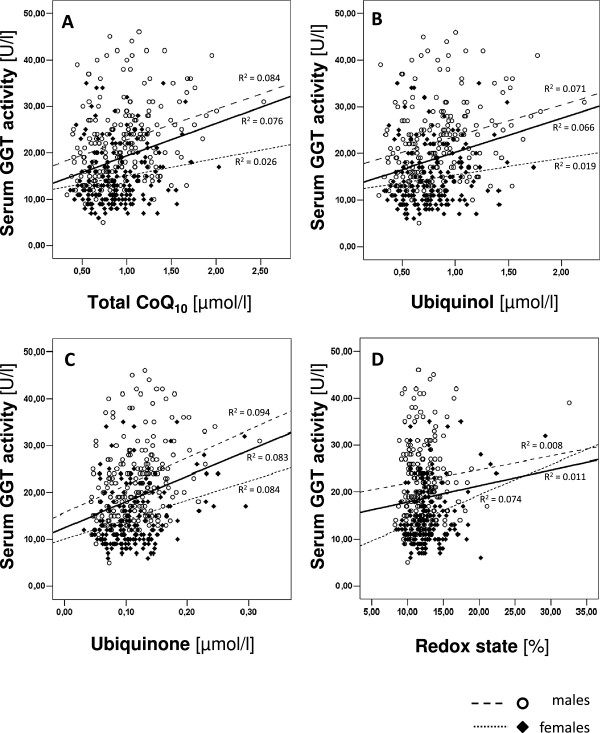
**Relationship between total CoQ**_**10 **_**(A), ubiquinol (B), ubiquinone (C) and CoQ**_**10 **_**redox state (D) and serum GGT activity.**

We conducted a correlation analysis to evaluate the relationship between CoQ_10_ status and serum GGT activity. As shown in Table 2, levels of total CoQ_10,_ ubiquinol, ubiquinone (oxidized form of CoQ_10_; all p < 0.001) and redox state (p < 0.05) were positively correlated with serum GGT activity in the whole study sample. Analyses conducted separately by gender revealed a stronger positive correlation between total CoQ_10_ level and serum GGT activity in men (p < 0.001) than in women (p < 0.05). Additionally, the reduced form of CoQ_10_, ubiquinol, was positively associated with serum GGT activity (p < 0.001) in men but not in women. In contrast, the oxidized form of CoQ_10_, ubiquinone, had a stronger positive correlation with serum GGT activity in women (p < 0.001) than in men (p < 0.01), and interestingly, there was a highly significant positive correlation between the redox state and serum GGT activity solely in women (p < 0.001). These results are shown in Figure 2, which depicts the positive linear association between CoQ_10_ status and serum GGT activity in the whole population as well as in gender-specific sub-groups. With the exception of the CoQ_10_ redox state, all parameters of CoQ_10_ status were positively correlated with serum GGT activity. However, there were gender-specific differences. While the data from men showed a stronger positive relationship between levels of both total CoQ_10_ (Figure 2A) and ubiquinol (Figure 2B) and serum GGT activity compared with the relationship found in women, there was only a small gender-related difference in the R^2^ of ubiquinone and serum GGT activity (Figure 2C). In addition, women but not men had a strong positive association between the redox state of CoQ_10_ and the activity of serum GGT activity (Figure 2D).

**Table 2:** **Correlation coefficients between Coenzyme Q**_**10 **_**(CoQ**_**10**_**) status and gamma glutamyltransferase (GGT)**

	**GGT**		
	**All (n = 383)**	**Males (n = 220)**	**Females (n = 196)**
**CoQ**_**10 **_**[μmol/l]**	0.276***	0.290***	0.161*
**Ubiquinol [μmol/l]**	0.258***	0.267***	0.137
**Ubiquinone [μmol/l]**	0.240***	0.217**	0.290***
**CoQ**_**10 **_**redox state [%]**	0.105*	0.089	0.273***

*p < 0.05; **p < 0.01, ***p < 0.001.

### CoQ_10_ is not associated with other liver-related enzymes (i.e., AST)

To evaluate the specificity of the relationship between CoQ_10_ and GGT, we also performed a correlation analysis with the parameters of CoQ_10_ status and serum aspartate aminotransferase activity (AST). As shown in Table 3, there were no significant associations between CoQ_10_ status and serum AST activity.

**Table 3:** **Correlation coefficients of Coenzyme Q**_**10 **_**(CoQ**_**10**_**) status and aspartate aminotransferase (AST)**

	**AST**		
	**All (n = 383)**	**Males (n = 199)**	**Females (n = 184)**
**CoQ**_**10 **_**[μmol/l]**	0.086	0.093	0.044
**Ubiquinol [μmol/l]**	0.097	0.099	0.062
**Ubiquinone [μmol/l]**	0.028	0.042	0.003
**CoQ**_**10 **_**redox state [%]**	-0.084	-0.105	-0.049

### Q_10_H_2_ Supplementation study – cohort II

To examine whether Q_10_H_2_ supplementation influences the activity of GGT and AST, we re-analyzed our human intervention study [27]. Within this study, 53 healthy male volunteers were supplemented with 150 mg ubiquinol per day for two weeks. Table 4 shows the basic characteristics of the study population (cohort II). The study subjects were between 21 and 48 years of age with a mean BMI of 24.1 ± 2.5 kg/m^2^. All parameters regarding CoQ_10_ status, CRP status as well as the parameters of metabolic syndrome defined by ATPIII were within a normal range.

**Table 4:** Basic characteristics of cohort II (supplementation study)

	**Males (n = 53)**
**Age [years]**	30.1 ± 6.7
**Body mass index [kg/m**^**2**^**]**	24.1 ± 2.5
**CoQ**_**10 **_**[μmol/l]**	0.96 ± 0.31
**Ubiquinol [μmol/l]**	0.89 ± 0.29
**Ubiquinone [μmol/l]**	0.07 ± 0.02
**CoQ**_**10 **_**redox state [%]**	7.5 ± 0.97
**Gamma GT [U/l]**	20.5 ± 10.4
**C-reactive protein [mg/dl]**	0.30 ± 0.01
**systolic BP [mm Hg]**	127 ± 12
**Diastolic BP [mm Hg]**	83 ± 9
**Triglycerides [mg/dl]**	97.4 ± 49.0
**Cholesterol [mg/dl]**	166.1 ± 29.8
**LDL-cholesterol [mg/dl]**	95.5 ± 28.9
**HDL-cholesterol [mg/dl]**	51.1 ± 12.9
**Glucose [mg/dl]**	86.5 ± 10.7

CoQ_10_ = Coenzyme Q_10_, Gamma GT = gamma glutamyltransferase; BP = blood pressure; LDL = low density lipoprotein, HDL = high density lipoprotein.

Data are presented as mean ± SD.

To investigate the effects of Q_10_H_2_ supplementation, fasting blood samples were taken before (T_0_) and after (T_14_) supplementation with Q_10_H_2_. As described previously, plasma CoQ_10_ levels increased more than fourfold from T_0_ to T_14_ (0.96 ± 0.31 pmol/μl to 4.60 ± 1.55 pmol/μl, p < 0.001, data not shown) whereas the redox state decreased significantly from 7.47 ± 0.97% to 5.95 ± 0.91% (p < 0.001; data not shown) [27].

### Q_10_H_2_ supplementation reduces serum GGT activity and mediates down regulation of GGT1 mRNA

**Figure 3:**
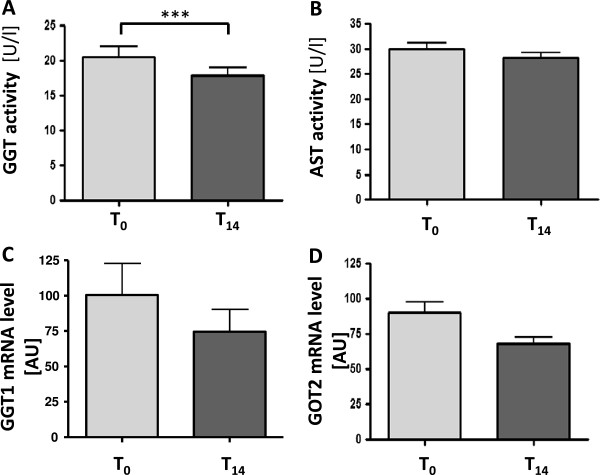
**The impact of Q**_**10**_**H**_**2 **_**supplementation on gamma glutamyltransferase (GGT, A) and *GGT1 *mRNA levels (B) as well as alanine aminotransferase (AST, C) and *GOT2 *mRNA levels (D).** Data show respective effects before (T_0_) and after (T_14_) supplementation. Data are calculated from the mean (±SEM) of 53 **(A + C)** or rather three **(B + D)** volunteers. Bar graphs are presented as the mean ± SD, ***p < 0.001 significant differences between T_0_ and T_14_, paired Student’s *t*-test.

Figure 3A shows a significant decrease of GGT activity from T_0_ to T_14_ (20.49 ± 10.36 U/l to 17.79 ± 7.68 U/l; p < 0.001). In contrast, we found no alteration of AST activity in response to Q_10_H_2_ supplementation (Figure 3B). To investigate whether the effect on GGT activity is also detectable on gene expression level, the mRNA steady state level of both *GGT1* (GGT) and *GOT2* (AST) were determined in CD14-positive monocytes of three subjects before and after Q_10_H_2_ supplementation. Indeed, we found that Q_10_H_2_ supplementation reduced the *GGT1* mRNA level with a fold change of -1.49 (Figure 3C) and the *GOT2* mRNA level with a fold change of -1.34 (Figure 3D).

## Discussion

### Association between CoQ_10_ status and serum GGT activity

Highly elevated serum GGT is a well-known marker of alcohol consumption, alcoholic liver diseases, and cholestasis [18]. Furthermore, several researchers have postulated that serum GGT activity, in its normal range, is a potential marker of oxidative stress [23-25]. It was shown in a longitudinal study that low circulating concentrations of several antioxidants (i.e., tocopherols, carotenoids) inversely predicted serum GGT concentrations ten years later [29]. Because CoQ_10_ is a known antioxidant, one would expect decreased levels of antioxidative CoQ_10_ or ubiquinol in the presence of high serum GGT activity. In the present study, there was a strong positive relationship between serum GGT activity and total CoQ_10_, ubiquinol and ubiquinone in study subjects with normal serum GGT activity. As we examined the relationship between CoQ_10_ status and serum GGT activity in a cross-sectional study, we were only able to examine one time point. Therefore, longitudinal studies should be conducted to elucidate the long-term relationship of serum CoQ_10_ and serum GGT activity. Nevertheless, it is possible that serum GGT may be an activator of the antioxidative system in humans exposed to oxidative stress. If so, CoQ_10_ might be the first antioxidant that is synthesized or released due to elevated serum GGT activity. This hypothesis is supported by ideas from the CARDIA study. There, it was speculated that an increase of serum GGT might eventually minimize oxidative stress by facilitating antioxidative defense (i.e., GSH) [23].

In summary, there is a strong relationship between human CoQ_10_ status and serum GGT activity. However, further investigations are needed to elucidate the complex relationship between antioxidant CoQ_10_ and serum GGT as a potential marker of oxidative stress in more detail. For this purpose, additional specific and stable byproducts of oxidative damage to lipids, DNA or proteins in the blood should also be examined in future research.

### Gender-specific differences in the association between CoQ_10_ status and serum GGT activity

It is known that higher CoQ_10_ levels as well as increased GGT activity are strongly associated with male gender [20,30,31]. This finding was confirmed in our study because men had higher levels of both CoQ_10_ and serum GGT activity than women. Additionally, this fact explains the gender-specific differences regarding the strength of the correlation between serum GGT activity and CoQ_10_ status in our study sample. The reasons for gender-related differences in CoQ_10_ levels and serum GGT activity are most likely physiologic and based on hormonal differences between the genders. It was previously shown in females that the use of oral contraceptives increased GGT activity level by 15 percent and pregnancy lowered GGT activity level by 25 percent, whereas the postmenopausal state was associated with a 7 percent increase in GGT activity [32]. Similar hormonal relationships have been shown for CoQ_10_ but in the opposite direction: women using oral contraceptives had lower levels of CoQ_10_ compared to women who were not using hormonal contraceptives [33]. This finding is in accordance with other studies that have shown increased oxidative stress in women using oral contraceptives [34]. Based on these findings, we could possibly explain the strong association of the CoQ_10_ redox state and serum GGT activity found in the women of our study. However, we do not know if our female study subjects were using oral contraceptives or other hormonal therapies. In future research, the hormonal status of female volunteers should be taken into account when investigating the relationship between serum CoQ_10_ levels and serum GGT activity in women.

### Relationship between serum CoQ_10_ status and serum AST

Similar to GGT, AST is associated with oxidative stress and increased all-cause mortality [35,36]. In contrast to GGT, we did not find an association between CoQ_10_ status and serum AST activity in our study. Therefore, we conclude that the association between GGT and CoQ_10_ is specific and does not appear to generalize to other liver-related enzymes such as AST.

### Supplementation study

#### The effect of Q_10_H_2_ supplementation on serum GGT/AST activity

CoQ_10_ is a known modulator of gene expression [4,5] and inflammatory processes [7-9]. Because the reduced form of CoQ_10_, ubiquinol, is a potent antioxidant that protects lipids, DNA and proteins from oxidative damage [12-14], we examined the effect of Q_10_H_2_ supplementation on both serum GGT and AST activity as potential markers of oxidative stress. Because we revealed an effect of Q_10_H_2_ supplementation on serum GGT activity but not on serum AST activity, we speculate that the effect of Q_10_H_2_ supplementation may be specific to serum GGT activity as a marker of oxidative stress in men. Support for this hypothesis comes from the strong associations found in cohort I between CoQ_10_ status and GGT activity but not between CoQ_10_ status and serum AST activity. In contrast, Yuvaraj *et al*. examined the effect of CoQ_10_ combined with riboflavin and niacin on serum GGT and serum AST in Tamoxifen-treated postmenopausal women with breast cancer [37] and found that the activity of both serum GGT and serum AST decreased in response to exogenous supplementation of CoQ_10_, niacin and riboflavin. Although Yuvaraj and colleagues used CoQ_10_ instead of ubiquinol and administered it in combination with niacin and riboflavin, the effect they found on serum AST might be due to a synergistic effect of all three antioxidative co-enzymes. Furthermore, they used a postmenopausal female study sample suffering from breast cancer whereas our study sample was male and healthy. Therefore, hormonal differences as well as disease status should be considered when examining the effects of Q_10_H_2_ supplementation on serum activity levels of liver-related enzymes (i.e., GGT, AST).

#### Effect of Q_10_H_2_ supplementation on GGT1/GOT2 mRNA level

Several studies have shown that oxidative stress, or rather instigators of oxidative stress (i.e., iron), lead to increased GGT mRNA as well as AST mRNA levels [35,38]. Here Q_10_H_2_ supplementation caused a transcriptional down regulation of *GGT1* mRNA and *GOT2* mRNA. The reduction in *GGT1* mRNA levels led to a decrease in serum GGT activity and a consequent decline in oxidative stress whereas the transcriptional down regulation of the *GOT2* gene caused no decline in serum AST activity.

In summary, Q_10_H_2_ reduced the activity of GGT as a marker of oxidative stress on protein and GGT1 mRNA level in CD14-positive monocytes. Indeed, there is a need for further studies examining the effect of Q_10_H_2_ supplementation on *GGT1* mRNA levels in liver tissue. Nevertheless, our findings provide preliminary evidence that there might be an effect of ubiquinol supplementation on serum GGT activity. This effect might be caused by gene expression. Additionally, this supplementation effect seems to be specific to serum GGT activity, as supplementation did not affect the activity of other liver enzymes (i.e., AST).

## Conclusions

There is a strong association between human CoQ_10_ status and serum GGT activity. However, the strength of this association depends on gender and is more pronounced in men than women. In contrast, female but not male volunteers showed a strong association between the CoQ_10_ redox state and serum GGT activity. Therefore, further studies are needed to examine the relationship between antioxidative CoQ_10_ and serum GGT activity as a marker of oxidative stress in both males and females in more detail. Because Q_10_H_2_ supplementation led to a decrease in serum GGT activity as well as *GGT1 mRNA* levels, we provide preliminary evidence that ubiquinol supplementation might improve oxidative stress on protein and gene expression levels. Further studies are needed to examine this effect in a larger cohort. Additionally, the effect of ubiquinol supplementation on *GGT1* mRNA levels should be further examined in organ tissue samples (i.e., liver).

## Competing interests

The authors declare that they have no competing interest regarding the publication of this article.

## Authors’ contributions

SO analyzed the data, interpreted results, and drafted and wrote the manuscript. PN and TM carried out the CoQ_10_ measurements. GJ, UN and WL were responsible for biobanking in data analysis. FD carried out the gene expression experiments and was responsible for the concept and design of the study, and drafting of the paper. All authors read and critically revised the final manuscript.
